# Cost of the typhoid conjugate vaccine introduction through an integrated campaign and follow-on routine immunization in Malawi

**DOI:** 10.1016/j.jvacx.2024.100583

**Published:** 2024-11-13

**Authors:** Frédéric Debellut, George Bello, Mike Chisema, Rouden Mkisi, Moses Kamzati, Clint Pecenka, Emmanuel Mugisha

**Affiliations:** a Center for Vaccine Innovation and Access, PATH, Geneva, Switzerland; b Epidemiology Unit, Ministry of Health, Lilongwe, Malawi; c Expanded Programme on Immunization, Ministry of Health, Lilongwe, Malawi; d Center for Vaccine Innovation and Access, PATH, Lilongwe, Malawi; e The Mitchel group, USAID Malawi Learn to Perform, Lilongwe, Malawi; f Center for Vaccine Innovation and Access, PATH, Seattle, WA, USA; g Center for Vaccine Innovation and Access, PATH, Kampala, Uganda

**Keywords:** Immunization delivery cost, Integrated campaign, Immunization campaign, Typhoid conjugate vaccine, Malawi

## Abstract

Malawi introduced typhoid conjugate vaccine (TCV) in 2023 through an integrated campaign delivering TCV alongside other vaccines and interventions (measles rubella vaccine (MRV), bivalent oral polio vaccine (OPV), and vitamin A Supplementation). The campaign sought to reach all children 9 months to younger than 14 years, representing more than 9 million individuals, and about half the country’s population. Following the campaign, TCV was incorporated into the routine immunization program for 9-month-old infants. We conducted a micro-costing study at 50 randomly selected health facilities, 10 districts, and at national level to retrospectively assess the financial and economic cost of the integrated campaign, as well as prospectively estimate the cost of delivering TCV in the routine immunization system. The financial and economic costs per dose for all interventions delivered in the campaign were $0.49 ($0.42;$0.57) and $0.84 ($0.67;$1.02), respectively. The main activities and cost types varied; human resources represented the main resource at health facility level, and per diem at district and national levels. The financial and economic cost to routinely deliver a dose of TCV were $0.44 ($0.17;$0.87), and $2.37 ($1.39;$3.53), respectively, with human resources as the main resource used by the routine program at all levels.

The cost per dose delivered in the integrated campaign in Malawi was comparable with other integrated campaigns and was lower than the reported cost to deliver TCV in single antigen campaigns in India and Zimbabwe. Integrated campaigns may represent an opportunity to introduce new vaccines such as TCV to lower the cost per dose delivered. Attention should be given to challenges coming with integration, such as the burden for healthcare workers.

Evidence produced by this study can be used in Malawi to inform financial sustainability of the TCV program and should inform decisions and strategies for implementation by other countries.

## Introduction

Typhoid represents a high burden in Africa and Asia [1]. Typhoid is estimated to be responsible for more than 7 million cases and 93,000 deaths worldwide annually, affecting mostly children and young adults from the poorest communities [2]. A surveillance study in Blantyre found an incidence of 444 per 100,000 person-years with a majority of the samples tested being of drug resistant strains [3]. The increase in drug resistant strains in Malawi is well documented, with multi drug resistant strains becoming prominent as early as 2011 [4], [5]. Recent passage of cyclone Freddy and its dramatic consequences is another reminder that the country is prone to extreme climate change weather related events, which has the potential to worsen the burden of water born disease such as typhoid [6].

Following the prequalification of the first typhoid conjugate vaccine (TCV) in 2017, the World Health Organization issued a recommendation for use of TCV in endemic areas and in countries with a high burden of drug resistant typhoid [7]. TCV represents a powerful tool alongside other available interventions to control typhoid. By mid-2024, 6 countries introduced TCV in routine immunization programs, including Malawi [8].

In May 2023, Malawi introduced TCV through an integrated campaign delivering TCV in addition to the provision of other vaccines and interventions (measles rubella vaccine (MRV), bivalent oral polio vaccine (OPV), and vitamin A supplementation). The target population for TCV, the largest of the four interventions, included all children 9 months to younger than 14 years old, more than 9 million individuals, representing about half of the country’s population. According to administrative coverage data, the campaign reached more than 7 million children with one dose of TCV, achieving a coverage rate of 77 %. Higher coverage rates were achieved for the other interventions: 83.2 % for MRV, 86.6 % for bOPV, and more than 100 % for vitamin A [9]. Following the campaign, TCV was incorporated into the routine immunization program for children 9-months-old.

To inform the decision-making for TCV introduction in Malawi, we projected the potential cost of a TCV campaign and routine immunization using a standardized costing framework [10]. Among the countries that have used TCV, only a few have studied the cost to deliver the vaccine, and none were nationwide introductions [11], [12]. There is also a lack of evidence on the cost of immunization campaigns; there is even less evidence when it comes to integrated campaigns [13], [14], [15].

Considering the lack of empirical data on the cost to deliver TCV and the unique introduction in Malawi, we retrospectively assessed the cost to deliver the vaccine through an integrated campaign and estimated the cost to deliver through routine immunization. The objective was to generate evidence for use by policymakers in Malawi to inform immunization budgeting and in other countries in the region to inform the cost to introduce and deliver TCV. We also intended to add to the body of evidence available on the cost of immunization campaigns.

## Methods

### Study objectives and design

This study is a micro costing study aiming to assess the incremental cost to provide TCV in Malawi. We collected data on the resources used during the integrated campaign that took place in May 2023 to retrospectively assess the cost to deliver all interventions provided. We collected data on resources used by the routine immunization program during the year 2022, assuming the ongoing routine costs for other antigens in the program would not change with the addition of TCV, to prospectively estimate the cost to deliver TCV routinely. We also collected data on outcomes achieved by both the campaign and the routine immunization program, including the number of doses delivered for each intervention during the campaign, and the doses delivered for each vaccine in the routine immunization program for year 2022. All cost data were collected from the provider’s perspective with no tracking of the payer. We followed costing principles from recently published guidelines for immunization campaigns and routine immunization programs costing, adapting existing data collection tools [16], [17]. We aligned our methods with the principles outlined in the WHO-led consensus statement on vaccine delivery costing [18]. We collected costs for a numerous campaign and program activities (e.g. training, service delivery, supervision…) and cost types (e.g. per diem, fuel, maintenance and energy, human resources…) as described in Table 1. We evaluated both financial and economic costs. While financial costs include direct expenditures at the time of the campaign or program, economic costs capture a broader perspective of costs, factoring in the opportunity cost of resources used that would have otherwise served another purpose, in addition to financial costs [16]. We did not include the cost of vaccines, vitamin A tablets, syringes, or safety boxes in the analysis.

**Table 1:** Program activities and cost types included in the study.

Program activities	Cost types	
	Financial cost	Opportunity cost
Estimating demand*	Meeting and events	Human resources
Training	Fuel, maintenance, and energy	Capital costs for vehicles and equipment
Campaign management or Program planning and management for routine immunization	Vehicle rental and transportation	
Social mobilization	Printing stationeries and communications	
Vaccine distribution and storage	Per diem	
Vaccine service delivery	Supplies***	
Supervision		
Waste management		
AEFI management**		
Record keeping and monitoring		

*Only for routine immunization costing.

** Adverse event following immunization, only for integrated campaign costing.

*** Supplies exclude vaccine or vitamin A costs, syringes, and safety boxes.

### Study area, sampling, and data collection

Malawi health system is divided into 5 health zones, 29 districts, and more than 800 health facilities. Health zones are involved in the storage and distribution of routine vaccines while districts, and the central level, are also in charge of immunization program planning, monitoring, and supervision. We included in our sampling frame all facilities that reported providing immunization services for the year 2022, independent of their ownership (public-government run, faith-based, private for profit or not for profit). We performed stratified random sampling to select a total of 50 health facilities in 3 purposively selected zones, from 10 different districts, to cover the different geographies of the country. Sampling was done using the Immunization Costing Sample Design Optimizer Tool, health facilities were weighted according to their target population size, using the number of first doses of MRV given in 2021 as a proxy for size [19].

We collected data using separate costing questionnaires for the campaign and the routine immunization program with different questionnaires per level. Data were collected electronically using Kobo Collect in July 2023. To reduce recall bias, we wanted to ensure data collection took place no later than 2 months after the end of the integrated campaign. Data were collected from the EPI offices and vaccine stores at the central and district levels, from the vaccine stores at the zone level, and from the 50 health facilities. As zones had no roles in the integrated campaign, vaccines and supplies were directly distributed from the central level to the districts, we only collected routine immunization data at that level. Because campaigns are known to be intensive in labor and since the plan was to concomitantly deliver 4 interventions to a large target population (about half the country population) during a short period of time, we incorporated questions about overtime and hiring of personnel specifically for the campaign to understand the implications of such an effort for the healthcare workforce.

Data collection included both primary and secondary data. Secondary data included the number of doses delivered as part of the campaign and the routine program as well as unit costs, equipment prices, and government staff salary scale. Secondary data used in the analysis are available from supplementary files.

Data analysis was done using Stata software (Stata v17, Stata Corp, TX, USA). For each resource, quantities were multiplied by their unit costs, then summed per activity and per cost type. This was done for each facility, each district, each zone, and the central level.

### Cost considerations

All data were collected in Malawian Kwacha (MWK), the currency of Malawi. Costs were converted to USD using the official World Bank currency exchange rate for year 2020 (1US$ = 749.53MWK) [20]. Capital costs were depreciated based on their respective Useful Life Years (ULY) and using a discount rate of 3 % (supplementary files).

At each level of the health system, we calculate and report for both the campaign and the routine immunization program the weighted average financial and economic cost per program activity, per cost type, and per dose delivered. For the campaign, the cost per dose reported is for all interventions delivered in the campaign.

For the routine immunization program, we calculated the cost for all vaccines delivered by the program in 2022. We summed costs for meetings, per diem, supplies, and human resources and divided this cost by the number of vaccine doses delivered to obtain a cost per dose. For vehicle rental and transportation, fuel, energy, and maintenance, and capital equipment, we divided the cost by the volume of vaccines delivered by the program in 2022 to obtain a cost per cm3 which we multiplied by the volume of a dose of TCV to obtain a cost per dose for TCV. We then added the cost per dose for each activity to obtain the cost to deliver TCV in the routine immunization program.

### Scenario analysis

As the campaign activities were fully integrated, it was not possible to disaggregate the cost per intervention to understand the cost to deliver TCV only. To explore what could have been the cost to deliver TCV in a standalone campaign, we made assumptions to adjust cost data, to reflect breadth and frequency of activities corresponding to a single intervention. To account for uncertainty, we developed a base case, and low and high scenarios in discussion with the expanded program on immunization team. These additional scenarios are not meant to reflect full details of potential variances in integration but rather to put bounds around the potential cost estimates. In the base case scenario, we adjusted cost according to the quantity of TCV doses delivered for activities related to training, campaign management, social mobilization, service delivery, waste management, supervision, AEFI management and record keeping, and monitoring. We calculated the cost of vaccine distribution and storage as a proportion to the volume of doses delivered. In the low scenario, we account for 25 % of the cost for all activities while maintaining a similar assumption for the base case for service delivery, vaccine distribution, and storage. For the high scenario, we account for 100 % of the cost for all activities while maintaining similar assumptions to the base case for service delivery and waste management. Detailed assumptions made per scenario are available in supplementary files. For each scenario, we calculated the cost per dose delivered, using the number of TCV doses delivered in the integrated campaign as a denominator.

### Ethical considerations

This study was reviewed by the PATH Research Determination Committee and was determined as research with no involvement of human subjects. In Malawi, the study protocol received ethical approval from the National Health Sciences Research Committee. Oral consent was obtained from all study respondents.

## Results

### Cost of the integrated campaign

We present the financial and economic cost of the integrated campaign along with the activities and cost types representing the largest share of total cost in Table 2. Detailed matrices of all costs per level, per activity, and per cost type are available from supplementary files.

**Table 2:** Financial and economic costs and main activities and cost types for the Malawi integrated campaign (2020 US$).

	**Health facility level (n = 50)**	**District level** **(n = 10)**	**National level** **(n = 1)**
**Financial cost**	$301 (Weighted mean cost)	$219,417 (Mean cost)	$2,155,830
**Economic cost**	$7,601 (Weighted mean cost)	$224,898 (Mean cost)	$2,180,366
**Activities representing the largest share of cost**	Service delivery Social mobilization Record keeping	Service delivery Training	Campaign management Training
**Cost type representing the largest share of cost**	Human resources	Per diem	Per diem Printing, communications, and stationeries

At the health facility level, the weighted mean financial cost was $301, with the majority of financial cost (58 %) for service delivery. The weighted mean economic cost was $7,601 and was composed of service delivery cost (26 %), social mobilization (18 %), and record keeping cost (17 %). The main cost type at health facility level was human resources, representing 94 % of the economic cost.

During the integrated campaign, an average number of 29,677 doses were delivered by each health facility in our sample across all interventions provided (Table 3). The weighted mean financial and economic costs per dose at that level were $0.01 (95 % CI: $0.01;$0.02) and $0.35 (95 % CI: $0.25;$0.46), respectively, as shown in Table 4.

**Table 3:** Doses delivered for each intervention during the Malawi integrated campaign.

	**Health facility level (n = 50)** Average doses delivered	**District level (n = 10)** Average doses delivered	**Nationally** **(n = 1)** Total doses delivered
**TCV** (9 months to 15 years)	13,347	326,439	7,043,335
**MRV** (9 months to 5 years)	5,428	123,885	2,646,095
**bOPV** (< 5 years)	5,749	134,219	2,925,288
**Vit A** (6 months to 5 years)	5,153	127,513	2,620,670
**Total**	29,677	712,056	15,235,388

**Table 4:** Financial and economic cost per dose delivered in the Malawi integrated campaign (2020 US$).

	**Health facility level**	**District level**	**National level**	**All levels**
**Weighted mean financial cost** (95 % CI)	$0.01 ($0.01;$0.02)	$0.34 ($0.27;$0.41)	$0.14	$0.49 ($0.42;$0.57)
**Weighted mean economic cost** (95 % CI)	$0.35 ($0.25;$0.46)	$0.35 ($0.28;$0.42)	$0.14	$0.84 ($0.67;$1.02)

At district level, the mean financial cost was $219,417, with the majority of cost due to service delivery (72 %). Of note, the financial cost at district level includes all per diem paid to vaccinators who operated at health facility level. With the vaccinator’s per diem removed the mean financial cost was $67,098 and the majority of this cost consisted of training (65 %). The mean economic cost at district level was $224,898 ($72,525 without per diem to vaccinators) with service delivery representing 71 % of this cost when accounting for per diems paid to vaccinators and training representing 61 % of the economic cost when excluding per diems.

The ten districts involved in the study delivered an average of 712,056 doses, all interventions included. The mean financial and economic costs per dose at district level were $0.34 (95 % CI: $0.27;$0.41) and $0.35 (95 % CI: $0.28;$0.42), respectively (Table 4).

At the national level, the financial cost was $2,155,830, and the economic cost was $2,180,366. The main activity contributing financial and economic costs was campaign management (representing 32 % of both financial and economic costs). The major financial cost types were per diems (35 %); printing, communications, and stationery (25 %); and meeting costs (22 %). The national level delivered a total of 15,235,388 doses for all interventions. The financial and economic costs per dose at that level were $0.14 (Table 4).

**Fig. 1:**
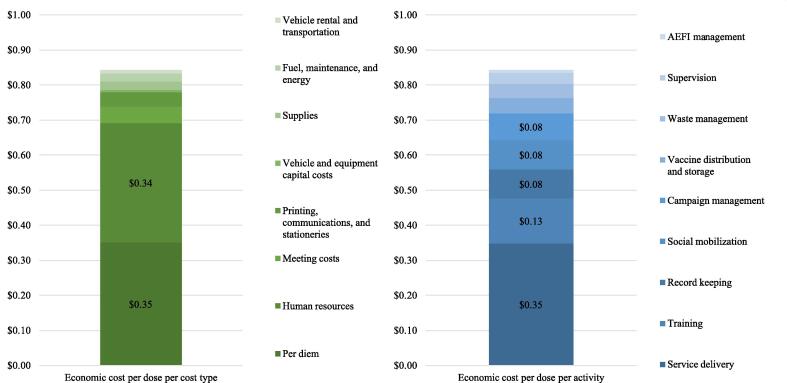
Economic cost per dose distribution per cost type and per activity (Malawi integrated campaign, 2020 US$).

When combining cost per dose at the different levels, the overall financial cost per dose in the integrated campaign was $0.49 (95 % CI:$0.42;$0.57), the overall economic cost per dose was $0.84 (95 % CI: $0.67;$1.02). Fig. 1 highlights the main activities and cost types comprising the economic cost per dose.

### Human resources implications for the integrated campaign

For health facilities included in the study, an average of 77 persons were involved in the campaign (min 4; max 338) with 17 staff, on average, hired specifically for the campaign (min 0; max 147). Four health facilities in the sample hired more than 100 additional staff specifically for the campaign. More than half of the sampled health facilities (52 %) reported longer working days during the campaign days with overtime spanning from half an hour to 4 h more per day, with an average of 1.8 h more per day. A smaller number of sampled health facilities (18 %) reported shorter days during the campaign days (on average shorter days by 1.2 h).

In the sampled districts, 13 persons, on average, were working on the campaign (min 4;max 32) with 8 % specifically hired for the campaign. At national level, 38 persons were involved in the campaign, and only 2 were hired specifically for the campaign.

### Scenario analysis: Cost to deliver TCV in a standalone campaign

**Fig. 2:**
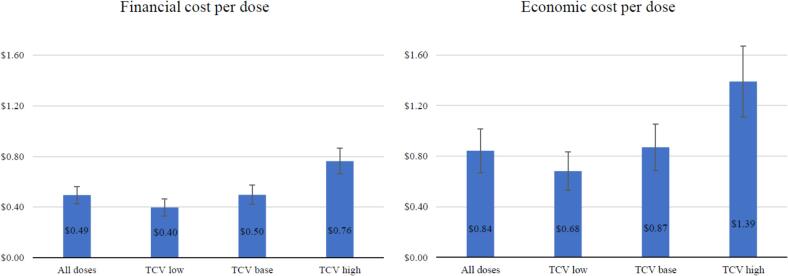
Financial and economic cost of TCV delivery as part of the Malawi integrated campaign (2020 US$).

Estimates from our scenario analysis show that the financial cost to deliver TCV in a standalone campaign range between $0.40 (95 % CI: $0.33;$0.46) in the low scenario and $0.76 (95 % CI: $0.66;$0.86) in the high scenario (Fig. 2). The economic cost per dose of TCV delivered ranges between $0.68 (95 % CI: $0.53;$0.83) in the low scenario and $1.39 (95 % CI: $1.11;$1.67) in the high scenario. In the base case scenario, the financial and economic cost to deliver TCV were $0.50 (95 % CI: $0.52;$0.47) and $0.87 (95 % CI: $0.69;$1.05) per dose, respectively.

### Cost to deliver TCV in the routine immunization program

The weighted mean financial cost of the routine immunization program at health facility level in 2022 was $7,384. The main activity driving the financial cost was waste management. The weighted mean economic cost was $26,290 and service delivery was the main activity. Mean financial and economic costs were higher at district, zone, and national levels and the main activity driving the cost was vaccine distribution and storage. The main cost type for the routine immunization program in 2022 at all levels was human resources. (Table 5).

**Table 5:** Financial and economic cost and main activities and cost types per level, Malawi routine immunization program (2020 US$).

	**Health facility level (n = 50)**	**District level** **(n = 10)**	**Zone level** **(n = 3)**	**National level** **(n = 1)**
**Financial cost**	$7,384 (Weighted mean cost)	$24,962 (Mean cost)	$16,796 (Mean cost)	$186,793
**Economic cost**	$26,290 (Weighted mean cost)	$53,197 (Mean cost)	$49,248 (Mean cost)	$408,502
**Main activity**	Waste management (fi) Service delivery (eco)	Vaccine distribution and storage	Vaccine distribution and storage	Vaccine distribution and storage
**Main cost type**	Human resources	Human resources	Human resources	Human resources

The estimated cost per dose to deliver TCV in the routine immunization system was higher at facility level. The weighted mean financial cost per dose at health facility level was $0.35 ($0.15;$0.54), and the weighted mean economic cost was $2.19 ($1.33;$3.04) (Table 6).

**Table 6:** Estimated financial and economic costs per dose of TCV delivered in the Malawi routine immunization program (2020 US$).

	**Health facility level** **(weighted cost)**	**District level**	**Zone level**	**National level**	**All levels**
**Mean financial cost** (95 % CI)	$0.35 ($0.15;$0.54)	$0.07 ($0.01;$0.31)	$0.00 ($0.00;$0.01)	$0.01	$0.44 ($0.17;$0.87)
**Mean economic cost** (95 % CI)	$2.19($1.33;$3.04)	$0.14 ($$0.02;$0.44)	$0.01 ($0.00;$0.02)	$0.03	$2.37 ($1.39;$3.53)

## Discussion

This micro-costing study shed lights on the resources used to deliver TCV and other interventions during the integrated campaign organized in Malawi in 2023, as well as the main resources used to run the routine immunization program. Human resources are a key resource supporting immunization in the country. It was the main economic cost type at the health facility level in the campaign and at all levels in the routine immunization program. For the integrated campaign particularly, the high number of staff hired specifically for the campaign and the high proportion of facilities where overtime was reported give an idea of the burden such an undertaking represents for health workers. Another key resource in the campaign was per diem, which was the main financial cost driver at district and national levels. This probably is a corollary of the large workforce required to carry out the campaign.

In the integrated campaign, the financial cost represented a very small proportion of the economic cost at health facility level, and conversely, a large proportion of the economic cost at district and national levels. This is illustrative of the rather centralized planning of the campaign with most spending taking place at these higher levels. It is also indicative of the labor-intensive activities at the health facility level.

The cost per dose delivered in the integrated campaign in Malawi is comparable to past (non-TCV) integrated campaigns that have been costed in Nigeria and Sierra Leone [21]. The cost per dose delivered in the integrated campaign in Malawi was lower than the cost to deliver TCV reported in single antigen campaigns in Navi Mumbai, India; and during an outbreak response in Zimbabwe [11], [12]. While we have not been able to cost integrated versus non-integrated delivery in Malawi since all health facilities in our sample delivered all four interventions, our scenario analysis tends to show that should TCV have been delivered standalone, the cost per dose would have likely been higher. More empirical evidence is required to capture the savings and level of economies of scale achieved by integrated immunization campaigns and demonstrate their benefits and challenges, including the potential burden on the health workforce it may represent.

The integrated campaign saw large contributions from Gavi. The financial cost per dose achieved remains below the amount that Gavi provides to cover campaign operational costs. This is likely due to the addition of vitamin A which was not subject to donor contributions.

The estimated financial cost to deliver TCV in the routine immunization system is similar to other studies conducted in Malawi, including the prior projection that assessed TCV introduction cost [10], [22], [23]. There was more variation of economic costs in studies previously conducted in Malawi, however, our estimate of the economic cost per dose fall within the range of what has been reported in the past. Our cost projections are subject to uncertainty and actual implementation of routine TCV may cost more or less, which could be assessed through future empirical studies. However, the scenario we explored is within the range of other existing estimates.

### Study limitations

This study had several limitations. First, the nationwide integration of the campaign in Malawi did not allow disaggregation of cost per intervention, nor did it allow for cost comparisons in areas with and without integration. To generate findings on cost to deliver standalone TCV we relied on scenario analysis. Second, per diem data, which represented a large share of the campaign cost, could not be consistently collected at health facility level for health workers operating at that level. We had to collect these data at district level to ensure completeness of our costing data. Third, the questionnaires were quite long and addressed two different activities with different time horizons. The questionnaire for the campaign, which had just occurred, was completed first, allowing for high quality data collection. Conversely, the routine activities occurred further in the past, and the questionnaire was completed after the campaign questionnaire, which resulted in a difference in the quality of data collected. Fourth, to estimate the routine cost to deliver TCV, we assumed that the ongoing routine costs for other antigens (i.e. MR, being delivered jointly) would not change with the addition of TCV, which may be optimistic considering TCV is a new vaccine and may not achieve similar coverage than other antigen already in the program in the year following introduction. We assumed introduction costs were incurred only during the introduction campaign.

## Conclusion

Malawi operated a successful campaign to introduce TCV while delivering 3 additional interventions in an integrated manner, at a favorable cost compared to other estimates in the literature, albeit still limited.. More research is required to fully understand potential economies of scale offered by integrated campaign delivery; however, one can assume that should the country have planned 4 campaigns instead of one, the total cost, as well as the burden on the healthcare workforce, would have likely been higher. This evidence should be used by decision-makers as they plan for upcoming immunization campaigns, as there may be an opportunity to introduce new vaccines such as TCV in an integrated manner. The benefits and challenges of integration should be weighed together. Future empirical studies can help validate our assumption that delivering TCV routine is likely to represent a cost similar to other routinely administered vaccines.

## CRediT authorship contribution statement

**Frédéric Debellut:** Writing – original draft, Visualization, Validation, Supervision, Software, Project administration, Methodology, Formal analysis, Conceptualization. **George Bello:** Writing – review & editing, Validation, Supervision, Software, Project administration, Methodology, Data curation. **Mike Chisema:** Writing – review & editing, Validation, Supervision, Methodology. **Rouden Mkisi:** Writing – review & editing, Validation, Supervision, Project administration, Methodology. **Moses Kamzati:** Writing – review & editing, Software, Data curation. **Clint Pecenka:** Writing – review & editing, Validation, Supervision, Methodology, Conceptualization. **Emmanuel Mugisha:** Writing – review & editing, Validation, Supervision.

## Declaration of competing interest

The authors declare that they have no known competing financial interests or personal relationships that could have appeared to influence the work reported in this paper.

## Data Availability

Data will be made available on request.
