# Supplementary material for: Cost of the typhoid conjugate vaccine introduction through an integrated campaign and follow-on routine immunization in Malawi
Source: Vaccine X. 2024 Nov 13;21:100583. doi: 10.1016/j.jvacx.2024.100583 (PMC11614825; doi:10.1016/j.jvacx.2024.100583)
Supplement: Supplementary Data 1 [file mmc1.pdf]

Cost of the typhoid conjugate vaccine introduction through an integrated campaign and follow-on routine immunization in Malawi

Supplements

Table 1. Quantities and respective volume of doses delivered during the integrated campaign for each intervention

Intervention	Volume per dose (in cm3)	Total doses delivered during the integrated campaign	Total volume of doses delivered during the integrated campaign
Typhoid conjugate vaccine	2.90	7,043,335	20,425,672
Measles rubella vaccine	3.90	2,646,095	10,319,771
Bivalent oral polio vaccine	0.94	2,925,288	2,749,771
Vitamin A	0.72	2,620,670	1,886,882

Table 2. Quantities and respective volume of vaccine doses delivered by the routine immunization program in 2022

Vaccine	Volume per dose (in cm3)	Total doses delivered by the routine immunization program in 2022
BCG	1.54	685,504
OPV	0.94	2,555,090
IPV	6.82	648,658
RVV	17.12	1,324,400
PCV	3.5	2,009,946
MR	3.9	1,096,884
HPV	15	57,868
PENTA	2.6	2,013,573
MAL	9.92	432,453

Table 3. Weighted mean integrated campaign cost matrix at health facility level

	Meeting cost	Fuel, maintenance, and energy	Vehicle rental and transportation	Printing, communications, and stationeries	Human resources	Vehicle and equipment capital cost	**Total**
Training	$8		$23	$0	$411	$1	**$443**
Service delivery	$1	$4	$169		$1,789	$14	**$1,976**
Campaign management	$31		$0	$10	$556	$0	**$598**
Supervision		$1	$0	$4	$179	$3	**$188**
Vaccine distribution and storage		$2	$1	$3	$711	$82	**$798**
Waste management		$1	$0	$0	$817	$3	**$820**
AEFI management					$140		**$140**
Record keeping				$37	$1,235	$13	**$1,285**
Social mobilization			$0	$4	$1,345	$2	**$1,350**
**Total**	**$40**	**$8**	**$194**	**$57**	**$7,183**	**$117**	**$7,599**

Table 4. Financial and economic cost of the campaign at health facility level

**Campaign activities**	**Weighted mean financial cost**		**Weighted mean economic cost**	
Training	$32	11%	$444	6%
Service delivery	$173	58%	$1,976	26%
Campaign management	$41	14%	$598	8%
Supervision	$6	2%	$188	2%
Vaccine distribution and storage	$6	2%	$798	11%
Waste management	$1	0%	$820	11%
AEFI management	-	0%	$140	2%
Record keeping	$38	13%	$1,287	17%
Social mobilization	$4	1%	$1,351	18%
**Total**	**$301**		**$7,601**	

Table 5. Mean integrated campaign cost matrix at District level

	Meeting cost	Fuel, maintenance, and energy	Vehicle rental and transportation	Printing, communications, and stationeries	Per diem	Human resources	Vehicle and equipment capital cost	**Total**
Training	$9,765	$631			$33,333	$489	$35	**$44,253**
Service delivery		$5,746			$152,373		$442	**$158,561**
Campaign management		$267		$197		$1,049	$5	**$1,519**
Supervision		$3,751		$11	$4,473	$1,395	$325	**$9,956**
Vaccine distribution and storage		$1,744		$6	$496	$542	$191	**$2,979**
Waste management		$140			$1,657	$145	$10	**$1,951**
AEFI management		$26				$3	$1	**$31**
Record keeping		$22		$29		$311	$133	**$495**
Social mobilization	$19	$185	$23		$4,575	$336	$15	**$5,153**
**Total**	**$9,784**	**$12,514**	**$23**	**$243**	**$196,907**	**$4,269**	**$1,158**	**$224,898**

Table 6. Financial and economic cost of the campaign at District level

Campaign activities	Mean financial cost		Mean economic cost	
Training	$43,729	20%	$44,253	20%
Service delivery	$158,119	72%	$158,561	71%
Campaign management	$464	0%	$1,519	1%
Supervision	$8,236	4%	$9,956	4%
Vaccine distribution and storage	$2,246	1%	$2,979	1%
Waste management	$1,797	1%	$1,951	1%
AEFI management	$26	0%	$31	0%
Record keeping	$51	0%	$495	0%
Social mobilization	$4,803	2%	$5,153	2%
**Total**	**$219,471**		**$224,898**	

Table 7. Mean integrated campaign cost matrix at district level (excluding per diems paid for health workers at health facility level)

	Meeting cost	Fuel, maintenance, and energy	Vehicle rental and transportation	Printing, communications, and stationeries	Per diem	Human resources	Vehicle and equipment capital cost	**Total**
Training	$9,765	$631			$33,333	$489	$35	**$44,253**
Service delivery		$5,746					$442	**$6,188**
Campaign management		$267		$197		$1,049	$5	**$1,519**
Supervision		$3,751		$11	$4,473	$1,395	$325	**$9,956**
Vaccine distribution and storage		$1,744		$6	$496	$542	$191	**$2,979**
Waste management		$140			$1,657	$145	$10	**$1,951**
AEFI management		$26				$3	$1	**$31**
Record keeping		$22		$29		$311	$133	**$495**
Social mobilization	$19	$185	$23		$4,575	$336	$15	**$5,153**
**Total**	**$9,784**	**$12,514**	**$23**	**$243**	**$44,534**	**$4,269**	**$1,158**	**$72,525**

Table 8. Financial and economic cost of the campaign at District level (excluding per diems paid for health workers at health facility level)

Campaign activities	Mean financial cost		Mean economic cost	
Training	$43,729	65%	$44,253	61%
Service delivery	$5,746	9%	$6,188	9%
Campaign management	$464	1%	$1,519	2%
Supervision	$8,236	12%	$9,956	14%
Vaccine distribution and storage	$2,246	3%	$2,979	4%
Waste management	$1,797	3%	$1,951	3%
AEFI management	$26	0%	$31	0%
Record keeping	$51	0%	$495	1%
Social mobilization	$4,803	7%	$5,153	7%
**Total**	**$67,098**		**$72,525**	

Table 9. Integrated campaign cost matrix at national level

	Meeting cost	Fuel, maintenance, and energy	Printing, communications, and stationeries	Supplies	Per diem	Human resources	Vehicle and equipment capital cost	**Total**
Training	$211,806	$3,325	$66,041		$276,295	$1,313	$432	**$559,213**
Service delivery				$255,932				**$255,932**
Campaign management	$188,089		$103,698	$103,698	$290,796	$4,029		**$690,311**
Supervision		$14,494			$54,382	$3,490	$2,162	**$74,527**
Vaccine distribution and storage		$2,712			$40,961	$2,195	$4,375	**$50,243**
Waste management								**$0**
AEFI management			$9,339			$456		**$9,795**
Record keeping			$330,775		$6,849	$176		**$337,800**
Social mobilization	$68,952	$1,641	$42,100		$83,945	$5,651	$258	**$202,545**
**Total**	**$468,847**	**$22,172**	**$551,953**	**$359,630**	**$753,228**	**$17,309**	**$7,227**	**$2,180,366**

Table 10. Assumptions used to inform scenarios exploring potential cost of TCV delivery as part of the integrated campaign

	**Low scenario**	**Base scenario**	**High scenario**
**Training**	Account for 1/4	Proportionate to quantity of TCV doses delivered in all doses/interventions delivered	100%
**Campaign management**	Account for 1/4	Proportionate to quantity of TCV doses delivered in all doses/interventions delivered	100%
**Social mobilization**	Account for 1/4	Proportionate to quantity of TCV doses delivered in all doses/interventions delivered	100%
**Vaccine distribution and storage**	Proportionate to volume of TCV doses delivered on all doses/interventions delivered for transportation<br>Proportionate to volume of TCV doses delivered on all vaccines delivered for cold chain storage		100%
**Service delivery**	Proportionate to quantity of TCV doses delivered in all doses/interventions delivered<br>Procurement related charges specific for TCV		
**Supervision**	Account for 1/4	Proportionate to quantity of TCV doses delivered in all doses/interventions delivered	100%
**Waste management**	Proportionate to volume of TCV doses delivered on all doses/interventions delivered		
**AEFI management**	Account for 1/4	Proportionate to quantity of TCV doses delivered in all doses/interventions delivered	100%
**Record keeping and monitoring**	Account for 1/4	Proportionate to quantity of TCV doses delivered in all doses/interventions delivered	100%

Table 11. Secondary data used in the analysis (MWK = Malawian Kwacha)

Replacement prices and useful life years (ULY) for equipment	
Mobile phone	30,000 MWK ULY 2 years
Smartphone	215,000 MWK ULY 2 years
Desktop	1,000,000 MWK ULY 5 years
Laptop	1,500,000 MWK ULY 5 years
Tablet	550,000 MWK ULY 2 years
Megaphone	150,000 MWK ULY 5 years
Printer	750,000 MWK ULY 5 years
Incinerator (health facility)<br>Incinerator (District and National)	150,000,000 MWK<br>300,000,000 MWK ULY 5 years
Toyota Landcruiser/4wd truck	$26,000 ULY 6 years
Ambulance	$30,500 ULY 6 years
Car	$15,000 ULY 6 years
Motorcycle	$3,500 ULY 4 years
Bike	150,000 MWK ULY 4 years
Generator (national level)	$25,304 ULY 5 years
Voltage regulator	65,000 MWK ULY 2 years
**Replacement prices and useful life years (ULY) for cold chain equipment**	
Cold box	$175 ULY 5 years
Ice pack	$3.50 ULY 2 years
Vaccine carrier	$1.8 - $34.9 depending on size ULY 5 years
Refrigerators and cold rooms	As per model and UNICEF pricelist ULY 10 years
Solar panel	317,500 MWK ULY 5 years
Solar inverter	700,000 MWK ULY 5 years
Air conditioner	1,106,750 MWK ULY 5 years
Fridge tag	$90 ULY 10 years
